# Substrate specificity of the MUS81-EME2 structure selective endonuclease

**DOI:** 10.1093/nar/gkt1333

**Published:** 2013-12-25

**Authors:** Alessandra Pepe, Stephen C. West

**Affiliations:** London Research Institute, Cancer Research UK, Clare Hall Laboratories, South Mimms, Herts EN6 3LD, UK

## Abstract

MUS81 plays important cellular roles in the restart of stalled replication forks, the resolution of recombination intermediates and in telomere length maintenance. Although the actions of MUS81-EME1 have been extensively investigated, MUS81 is the catalytic subunit of two human structure-selective endonucleases, MUS81-EME1 and MUS81-EME2. Little is presently known about the activities of MUS81-EME2. Here, we have purified MUS81-EME2 and compared its activities with MUS81-EME1. We find that MUS81-EME2 is a more active endonuclease than MUS81-EME1 and exhibits broader substrate specificity. Like MUS81-EME1, MUS81-EME2 cleaves 3′-flaps, replication forks and nicked Holliday junctions, and exhibits limited endonuclease activity with intact Holliday junctions. In contrast to MUS81-EME1, however, MUS81-EME2 cuts D-loop recombination intermediates and in so doing disengages the D-loop structure by cleaving the 3′-invading strand. Additionally, MUS81-EME2 acts on 5′-flap structures to cleave off a duplex arm, in reactions that cannot be promoted by MUS81-EME1. These studies suggest that MUS81-EME1 and MUS81-EME2 exhibit similar and yet distinct DNA structure selectivity, indicating that the two MUS81 complexes may promote different nucleolytic cleavage reactions *in vivo*.

## INTRODUCTION

The MUS81 protein is required for the maintenance of genomic stability, and its loss has been associated with cancer development (1,2). MUS81 is the catalytic subunit of the MUS81-EME1 structure-selective endonuclease that plays important roles in DNA repair, including (i) the repair of interstrand cross-links (3), (ii) the repair and restart of stalled replication forks (4–7) and (iii) the resolution of recombination intermediates (8–11). In both yeast and mammalian cells, loss of MUS81 activity leads to a hypersensitivity to replication fork-stalling agents such as cisplatin, camptothecin or hydroxyurea (2,3,12,13). MUS81 is also required for telomere maintenance in cells that use an Alternative Lengthening of Telomeres (ALT) telomerase-independent mechanism for telomere maintenance (14).

In addition to the mitotic functions, MUS81 is important for meiosis: for example, in yeast, Mus81-Eme1 (*Schizosaccharomyces pombe*) and Mus81-Mms4 (*Saccharomyces cerevisiae*) are required for the resolution of meiotic recombination intermediates (15–20). Similarly, *Mus81*-deficient mice exhibit defects in the repair of meiotic double strand breaks and reduced numbers of mature epididymal sperm (21). Purified recombinant Mus81-Eme1 and Mus81-Mms4 proteins are active on a range of DNA substrates including 3′-flaps, replication forks and nicked Holliday junctions (HJs), which they cleave by the introduction of a nick close to the branch point (22–25). In contrast, intact HJs are cleaved with a relatively low efficiency. Recombinant human MUS81-EME1 exhibits similar substrate specificities (9,26,27).

Recent studies have shown that *S. cerevisiae* Mms4 is phosphorylated in a cell cycle-dependent manner by the cyclin-dependent kinase Cdk and the Polo-like kinase Cdc5 leading to a stimulation of Mus81-Mms4 activity (28–31). Similarly, in human cells, phosphorylation of EME1 by CDK, and to a lesser extent by PLK1, correlates with increased MUS81-EME1 nuclease activity at prometaphase (9,28). In contrast to yeast, however, phosphorylation does not directly activate the nuclease activity of the enzyme but promotes an interaction between MUS81-EME1 and a second structure-selective nuclease SLX1-SLX4 (9). The formation of a MUS81-EME1-SLX1-SLX4 complex appears to be important for Holliday junction resolution, especially in the absence of BLM, and is critical for proper chromosome segregation (9–11). Hence, *S. cerevisiae* Mms4 and human EME1 appear to be the regulatory subunits of the Mus81-Mms4 and MUS81-EME1 endonucleases, respectively.

In addition to its interaction with EME1, human MUS81 can also interact with the EME2 protein. EME2 was identified by its sequence similarity with EME1, with the highest homology observed in the C-terminal domain (26). MUS81, EME1 and EME2 are all members of the XPF/MUS81 family of proteins and contain an ERCC4 endonuclease domain and a helix-hairpin-helix (HhH)_2_ domain (32). The ERCC4 domains of EME1 and EME2, however, have diverged in amino acid sequence, thus making these subunits catalytically inactive. No yeast orthologue of EME2 has been identified, indicating that MUS81-EME2 promotes reactions that are specific to higher eukaryotes.

Preliminary studies carried out with recombinant MUS81-EME1 and MUS81-EME2 purified from *Escherichia coli* showed that MUS81-EME2 exhibits 10-fold greater nucleolytic activity than MUS81-EME1 on a 3′-flap substrate (33). However, whether MUS81-EME2 exhibits any novel structure-selective endonuclease activities was not investigated. In this work, we have performed a comparative biochemical analysis of human MUS81-EME1 and MUS81-EME2 following their purification from baculovirus-infected insect cells. We find that MUS81-EME2 is a more active endonuclease than MUS81-EME1 and exhibits broader substrate specificity.

## MATERIALS AND METHODS

### Proteins

MUS81-_SF_EME1, MUS81-_SF_EME2 and MUS81^D307A^-_SF_EME2 were purified from insect cells following their expression from the baculovirus vectors pFL-MUS81-_SF_EME1, pFL-MUS81-_SF_EME2 and pFL-MUS81^D307A^-_SF_EME2, respectively. In brief, 600 ml of Hi5 cells (at 1 × 10^6^ cells/ml) were infected with the indicated baculovirus for 72 h. Cells were harvested by centrifugation at 3000 rpm, washed in ice-cold phosphate-buffered saline and resuspended in 30 ml of TGN buffer (20 mM Tris–HCl, pH 7.5, 10% glycerol, 0.01% NP-40) supplemented with 0.5 M NaCl, protease inhibitor cocktail (Roche), phosphatase inhibitor cocktail 2 (Sigma) and 1 mM dithiothreitol. Cells were lysed on ice for 45 min and homogenized with a Dounce pestle B (20 strokes). The lysate was ultracentrifuged for 45 min at 35 000 rpm (Beckman Type 45 Ti rotor), and the clarified extract was loaded overnight (0.2 ml/min) on a 1 ml Strep-Tactin Superflow column using a ÄKTAprime plus chromatography system (GE Healthcare) at 4°C. The column was washed with 20 column volumes of TGN buffer (0.5 ml/min) containing 0.5 M NaCl, and proteins were eluted with the same buffer supplemented with 2.5 mM desthiobiotin (40 × 0.5 ml elution fractions, 0.5 ml/min). Eluted proteins were identified by sodium dodecyl sulphate–polyacrylamide gel electrophoresis (SDS–PAGE). Peak fractions were pooled and loaded onto a 1 ml anti-FLAG M2 column (Sigma) for 2 h at 4°C. The column was washed with 20 column volumes of TGN buffer containing 0.5 M NaCl, and proteins were eluted in the same buffer containing 500 μg/ml of 3X FLAG peptides. Eluted proteins were identified by SDS-PAGE and diluted to 100 mM NaCl before loading onto a 1 ml HiTRAP heparin column (GE Healthcare) at 0.5 ml/min. The column was washed with 30 ml of TGN buffer containing 100 mM NaCl and proteins eluted using a 30 ml linear salt gradient (100 mM–1 M NaCl). Fractions containing MUS81-_SF_EME1, MUS81-_SF_EME2 or MUS81^D307A^-_SF_EME2A were dialyzed against storage buffer (50 mM Tris–HCl, pH 8.0, 10% glycerol, 100 mM NaCl and 1 mM dithiothreitol) and stored at −80°C. The final yields were MUS81-_SF_EME1 (15 μg), MUS81-_SF_EME2A (24 μg) and MUS81^D307A^-_SF_EME2 (9 μg).

GEN1^1^^−^^527^ was purified as described (34).

### Nuclease assays

Synthetic DNA substrates were prepared by annealing gel-purified oligonucleotides (35). The sequences are described elsewhere (9). The D-loop structure was prepared by annealing the following oligonucleotides:

DL-0: 5′-CGTTGGACGCTGCCGAATTCTACCACTGCGTGCCTTGCTAGGACATCTTTGCCCACCTGCAGGTTCACCCATCGC-3′
DL-1: 5′-GCGATGGGTGACCTGCAGGTGGGCGGCTGCTCATCGTAGGTTAGTGAATTGGTAGAATTCGGCAGCGTCCAACG-3′
DL-2: 5′-GATCGTAAGAGCAAGATGTTCTATAAAAGATGTCCTAGCAAGGCACGCAG-3′
DL-3: 5′-TATAGAACATCTTGCTCTTACGATC-3′

Reactions (10 μl) contained 100 nM of cold DNA, supplemented with 1 μl of 5′-^32^P-end-labeled DNA and cleavage buffer [50 mM Tris–HCl, pH 8.0, 3 mM MgCl_2_, 1 mM dithiothreitol and 100 µg/ml bovine serum albumin). Proteins to be analysed were diluted in 50 mM Tris–HCl, pH 8.0, 10% glycerol, 100 mM NaCl, 1 mM dithiothreitol and 100 µg/ml bovine serum albumin. Unless indicated otherwise, incubation was for 30 min at 37°C. Reactions were stopped by addition of one-fifth volume of stop buffer (100 mM Tris–HCl, pH 7.5, 50 mM EDTA, 2.5% SDS and 10 mg/ml proteinase K). Reaction products were analysed by neutral or denaturing PAGE, using appropriate markers, followed by autoradiography or phosphorimaging.

### Amplification of EME2 isoforms

RNA was extracted from the human breast carcinoma cell line MCF-7 using the RNeasy kit (Qiagen), according to the manufacturer’s instructions, and complementary DNA (cDNA) synthesis was performed using Illustra Ready-To-Go RT-PCR beads (GE Healthcare). EME2 isoforms were amplified by PCR using KOD Hot Start DNA polymerase (EMD Millipore). PCR products were analysed by agarose gel electrophoresis.

### Cellular expression and immunoprecipitation of _SF_EME2 and _SF_EME2B

T-REx HEK293 cells were transfected with pcDNA4-TO-_SF_EME2 or pcDNA4-TO-_SF_EME2B DNA, and stable clones were selected. Expression of _SF_EME2 or _SF_EME2B was induced by treatment with 1 μg/ml tetracycline for 72 h. The cells were then washed with phosphate-buffered saline, treated with trypsin and harvested by centrifugation. The cell pellets were resuspended in 500 μl of lysis buffer (30 mM Tris–HCl, pH 7.4, 150 mM NaCl) supplemented with phosphatase and protease inhibitor cocktails, and 0.5% NP-40. The DNA was sheared by passage through a 0.8 × 40 mm needle (20×), followed by incubation on ice for 30 min and centrifugation for 30 min at 14 000 rpm at 4°C. Cleared lysates were transferred to a fresh tube, and the protein concentrations were quantified using a Bio-Rad *D_c_* protein assay kit. The _SF_EME2 proteins were immunoprecipitated using anti-FLAG M2 resin and, after extensive washing, eluted with 3X FLAG peptides. Proteins were analysed by SDS–PAGE and detected by western blotting using mouse anti-FLAG M2-HRP antibody (Sigma A8592, diluted 1:500). MUS81 protein was visualized after western blotting with a monoclonal MUS81 antibody (Abcam ab14387, 1:500).

### The siRNA treatment

The EME2 siRNA (5*′*-GCGAGCCAGUGGCAAGAGA-3′) was purchased from Eurofins. One day before transfection, 7 × 10^5^ cells (HeLa, GM847 or U2OS) were seeded in 10-cm cell culture plates and then transfected with 80 nM EME2 siRNA using Lipofectamine® RNAiMAX. As a control, luciferase GL2 siRNA was used. Cells were collected 72 h after transfection and extracts analysed by western blotting.

The rabbit EME2 antibody (APEP13) was raised against a mixture of four peptides (amino acids 1–13, 13–36, 208–218 and 401–418) and affinity purified using protein A agarose.

### Immunofluorescence analysis

For immunofluorescence analysis, U2OS cells were transfected with pcDNA4-TO-_SF_EME2 DNA, and stable clones were selected. The _SF_EME2 expression was induced by treatment with 1 μg/ml tetracycline for 24 h. As required, the cells were treated with cisplatin (20 µg/ml, 5 h). The cells were fixed and processed for immunostaining essentially as described (36). The _SF_EME2 was visualized using rabbit anti-FLAG antibody (Sigma, F7425) and Alexa Fluor 488 (Molecular Probes, diluted 1:250). MUS81 was visualized using mouse anti-MUS81 antibody (Abcam ab14387, 1:500) and Alexa Fluor 546 (Molecular Probes, diluted 1:250). DNA was stained with DAPI. Images were acquired using a Zeiss AXIO Imager M1 with a 63× EC-Plan-Neofluor lens and Hamamatsu photonics camera under the control of Volocity software. Images were processed using Adobe Photoshop.

## RESULTS

### Two isoforms of EME2 are expressed in human cells

The human EME2 sequence was originally identified using a PSI-BLAST search for proteins orthologous to *S. pombe* EME1 (NCBI # XM_113869) (26,37). More recently, the EME2 sequence in the NCBI database was replaced by a sequence predicted to encode a 444 amino acid protein (NM_001010865). However, this sequence has also recently been revised to one that now encodes a 379 amino acid protein (NM_001257370.1). In addition to the sequence deletion, there are two amino acid changes compared with the earlier version.

**Figure 1.**
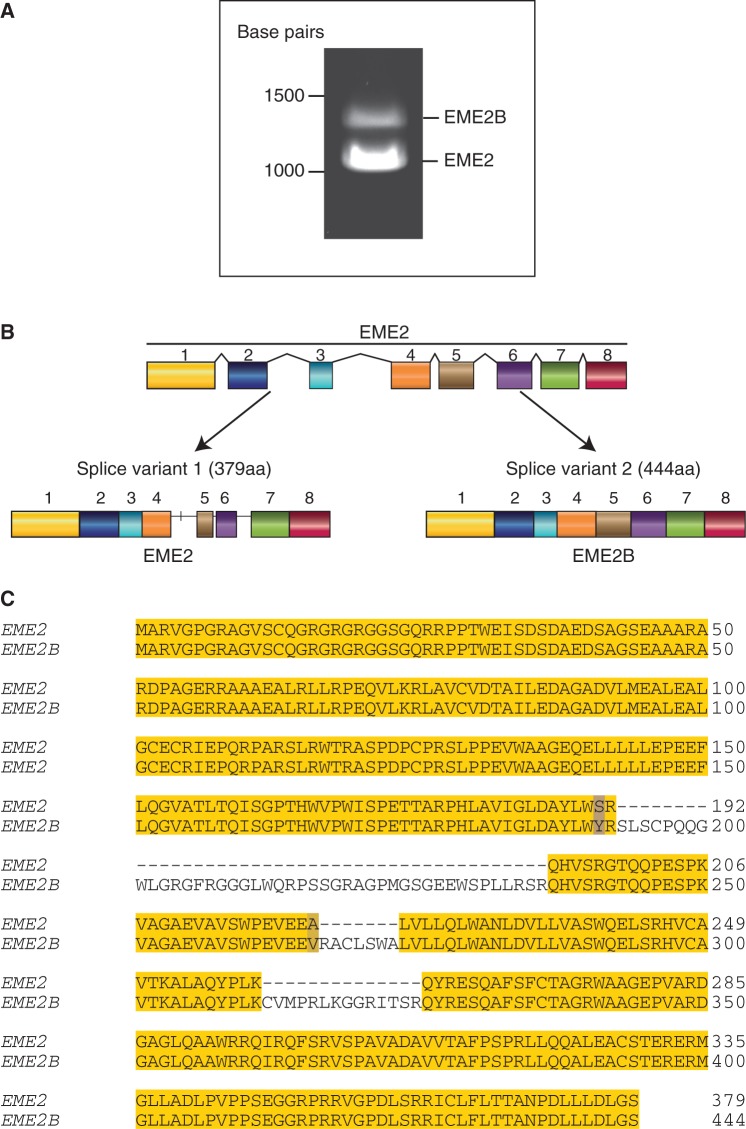
Expression of EME2 in human cells. (**A**). PCR amplification of the EME2 sequence from DNA extracted from human MCF-cells. DNA was visualized by agarose gel electrophoresis and staining with SafeView. (**B**). Schematic representation of the two isoforms of EME2, designated EME2 and EME2B. (**C**). Sequence alignment of the EME2 and EME2B proteins. Blue = non-conserved residues; yellow = conserved residues. Sequence alignments were made using ClustalW and Jalview.

To verify whether which, if any, transcript variants are expressed in human cells, the EME2 sequence was amplified from human breast adenocarcinoma MCF-7 cells. To do this, messenger RNA was extracted from MCF-7, and the cDNA was prepared and amplified by PCR, revealing two products of ∼1100 and 1300 bp. Sequence analyses revealed that the shorter more abundant product corresponded to the 379 amino acid EME2 protein (NM_001257370.1), whereas the longer sequence (NM_001010865) would encode a 444 amino acid protein, here designated EME2B (Figure 1A). These variants result from the alternative splicing of exons 4, 5 and 6 (Figure 1B).

**Figure 2.**
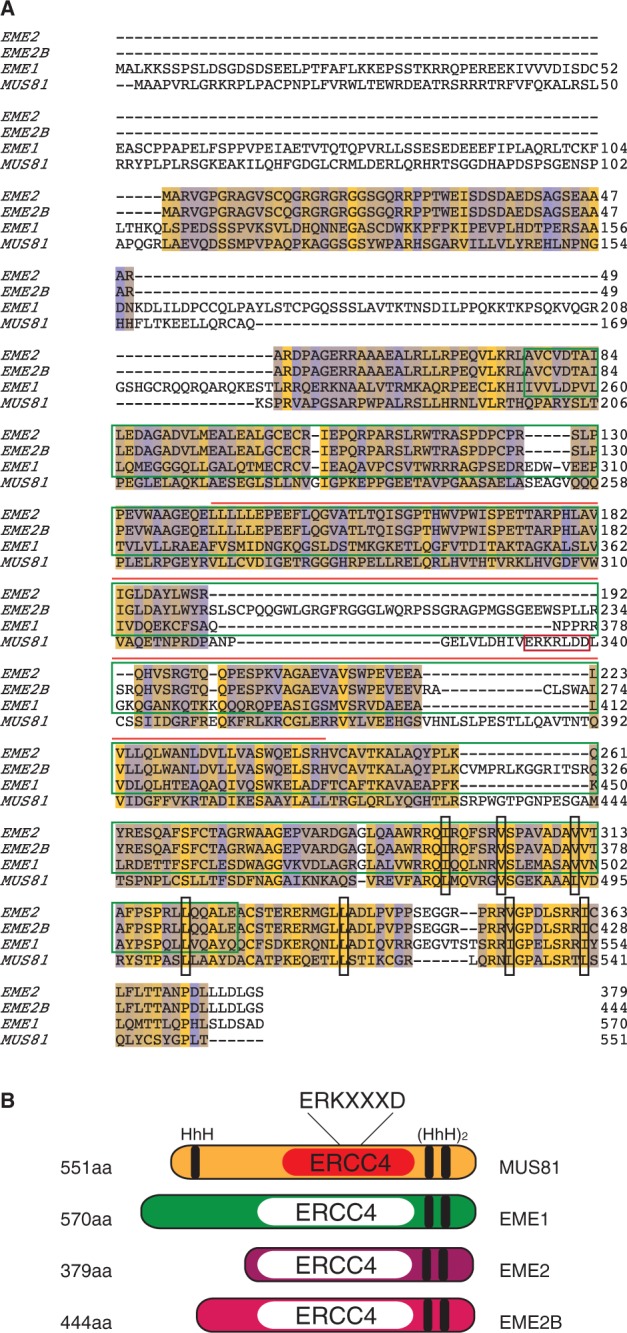
Sequence alignment between EME2, EME2B, EME1 and MUS81. (**A**). Conserved and non-conserved residues are indicated in yellow and blue, respectively. The ERKXXXD nuclease domain of MUS81 (red box), the ERCC4 domains of EME1, EME2 and EME2B (dark green box) and the ERCC4 domain of MUS81 (orange line) are indicated. Black boxes indicate important hydrophobic residues for the formation of the HhH domain. Sequence alignments were performed using ClustalW and analysed with Jalview. (**B**). Schematic representation of MUS81, EME1, EME2 and EME2B. HhH and ERCC4 nuclease domains are indicated in black. The active ERCC4 domain of MUS81 is indicated in red. Inactive ERCC4 domains are indicated in white.

Sequence alignment of EME2 and EME2B shows that the proteins exhibit 85.36% identity (Figure 1C). Human MUS81, EME1 and EME2 have a conserved C-terminal domain, through which they are believed to interact to form active endonuclease heterodimers (26). Sequence alignment of MUS81, EME1, EME2 and EME2B showed that the EME2 isoforms align mainly with the C-terminal region of MUS81 and EME1 (Figure 2A). EME1 shares 37% identity and 61.2% similarity with EME2 and 33.1% identity and 56.9% similarity with EME2B (Figure 2A). Both EME2 (residues 77–326) and EME2B (residues 77–391) contain an ERCC4 nuclease domain (Figure 2B) but, as already observed for EME1, the amino acid sequence of the ERKXXXD catalytic motif has diverged (compare amino acids 333–339 of MUS81 with the corresponding amino acids of EME1, EME2 and EME2B). Like MUS81 and EME1, EME2 and EME2B harbour a C-terminal (HhH)_2_ domain (32).

### Expression of EME2 and EME2B

**Figure 3.**
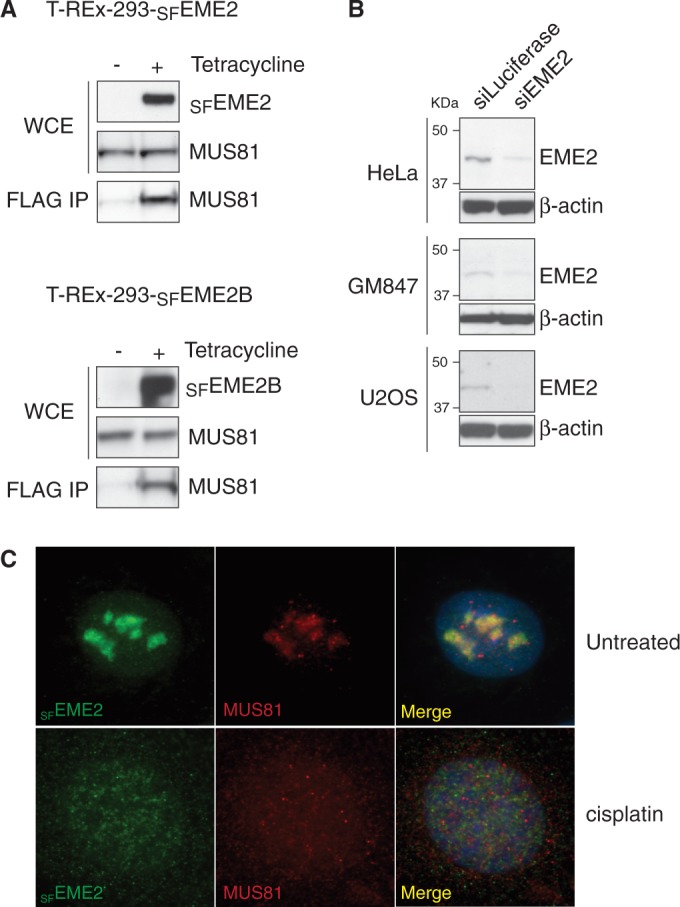
Interactions of MUS81 with EME2 and EME2B. (**A**). Extracts were prepared from T-REx-293 cells expressing _SF_EME2 or _SF_EME2B from a tetracycline-inducible promoter and probed by western blotting for the indicated proteins. The _SF_EME1 or _SF_EME2 proteins were immunoprecipitated using FLAG antibodies and the immunoprecipitates were probed for the presence of MUS81. (**B**). Western blots showing the presence of EME2 in the indicated cell lines, with and without siRNA treatment. Beta-actin was used as a loading control. (**C**). The U2OS cells expressing _SF_EME2 from a tetracycline-inducible promoter were treated with cisplatin. The _SF_EME2 and MUS81 proteins were detected by immunofluorescence using anti-FLAG and anti-MUS81 antibodies, respectively. DNA was visualized by DAPI staining.

To confirm that EME2 and EME2B interact with MUS81, streptavidin/FLAG-tagged EME2 (_SF_EME2) or EME2B (_SF_EME2B) was expressed in T-REx-293 cells using a tetracycline-inducible promoter. The proteins were then immunoprecipitated using anti-FLAG resin and probed for the presence of MUS81 by western blotting. We found that the _SF_EME2 and _SF_EME2B immunoprecipitates both contained endogenous MUS81 protein (Figure 3A). Given that we did not observe EME1 in these immunoprecipitates (data not shown), these results indicate that human MUS81 forms three distinct heterodimeric complexes: MUS81-EME1, MUS81-EME2 and MUS81-EME2B.

The distinct differences in transcript levels observed for EME2 and EME2B (Figure 1A) next led us to determine whether both proteins were expressed *in vivo*. To do this, an antibody was raised against a mixture of four peptides (amino acids 1–13, 13–36, 208–218 and 401–418) that would be capable of detecting endogenous EME2 and EME2B by SDS–PAGE and western blotting. When extracts of HeLa, GM847 and U2OS cells were analysed for the presence of the EME proteins, we observed only a single band that corresponded to the EME2 isoform (Figure 3B). This band was not observed, or observed at significantly lower levels, when the same cells were treated with siRNA against EME2. Given that we could find little evidence for the expression of EME2B (encoded by the minor transcript) in human cells, we focused our efforts on EME2. This 379 amino acid protein corresponds to the current NCBI database entry NM_001257370.1.

Attempts to determine the subcellular localization of endogenous EME2 by immunofluorescence analysis were unsuccessful. However, we were able to visualize _SF_EME2 after tetracycline-inducible expression in U2OS cells, finding that it localized to the nucleoli in untreated cells. Importantly, however, EME2 redistributed to the nucleoplasm following treatment with the DNA-damaging agents cisplatin (Figure 3C) or camptothecin (data not shown). The redistribution of EME2 in response to DNA damage is similar to that observed with MUS81 (Figure 3C) and implies a role for MUS81-EME2 in DNA repair. Further details of the repair role of EME2 will be presented elsewhere (Pepe,A. and West,S.C., in preparation).

### Substrate specificity of MUS81-EME2

**Figure 4.**
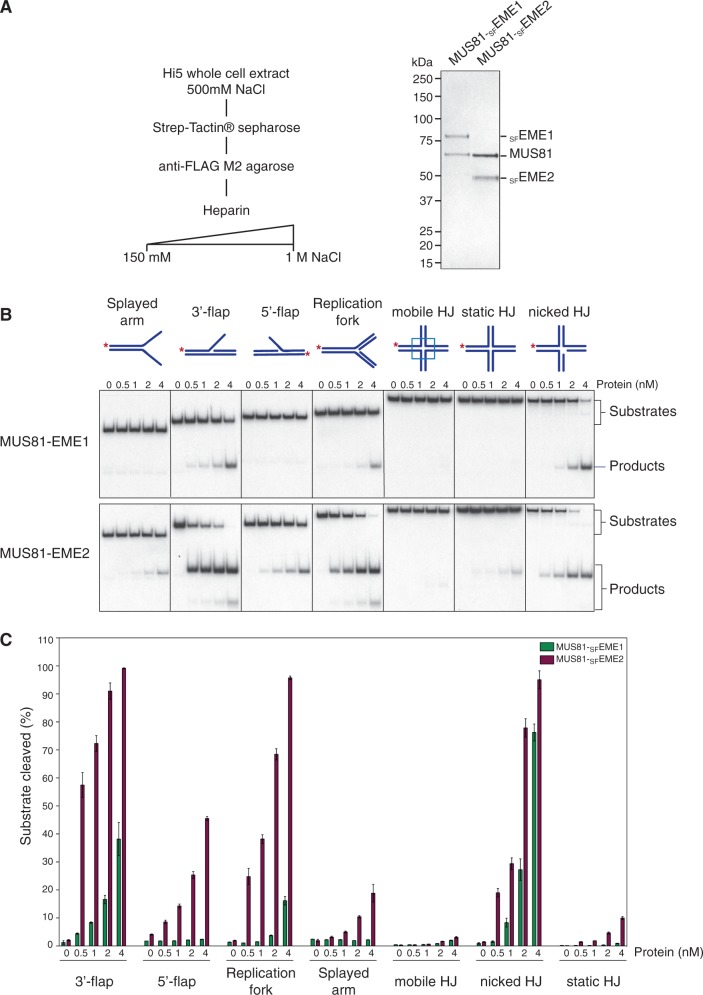
Substrate specificities of human MUS81-_SF_EME1 and MUS81-_SF_EME2. (**A**). Purification scheme and visualization of purified human MUS81-_SF_EME1 and MUS81-_SF_EME2 by SDS–PAGE followed by staining with InstantBlue. (**B**). The ^32^P-labelled DNA substrates (100 nM) were incubated with the indicated concentrations of purified MUS81-EME1 or MUS81-EME2 for 30 min at 37°C, and the products were analysed by neutral PAGE and visualized by autoradiography. The 5′-^32^P-end labels are indicated with asterisks. (**C**). Quantification of the data shown in Figure 4B was performed by phosphorimaging analysis. Product formation is expressed as a percentage of total radiolabelled DNA. Data are presented as the mean of three experiments (±SEM).

To determine the biochemical properties of MUS81-EME2, MUS81 and _SF_EME2 were coexpressed in insect cells from a bicistronic baculovirus vector. A similar construct was used to express MUS81-EME1 carrying the same affinity tags. The MUS81-_SF_EME1 and MUS81-_SF_EME2 proteins were then purified to homogeneity (Figure 4A) and analysed for their ability to cleave a variety of ^32^P-labelled DNA substrates containing secondary structures (Figure 4B). These included splayed-arm, 3′-flap, 5′-flap, replication fork and HJ structures, all containing a common 5′-^32^P-end-labelled oligonucleotide (X0.1). The HJs used in this analysis have a mobile homologous core (mobile HJ), an immobile non-homologous core (static HJ) or a nick adjacent to the branch point (nicked HJ).

MUS81-EME2, like MUS81-EME1, was found to act preferentially on the 3′-flap, replication fork and nicked HJ substrates. Only low levels of activity were observed with intact HJs. The activity of MUS81-_SF_EME2 was ∼5-fold greater than that of MUS81-_SF_EME1 on each substrate (Figure 4B and C). In contrast to MUS81-EME1, however, MUS81-EME2 was also able to cleave the 5′-flap structure. The ability to cleave both 3′- and 5′-flap structures is reminiscent of the activities of SLX1-SLX4 (9) but unusual for a member of the XPF-ERCC1 nuclease family (32). Moreover, we observed that the nicked/gapped duplex products resulting from flap cleavage were further processed into fast-migrating half-length duplexes (Figure 4B). These products were not observed with MUS81-EME1.

**Figure 5.**
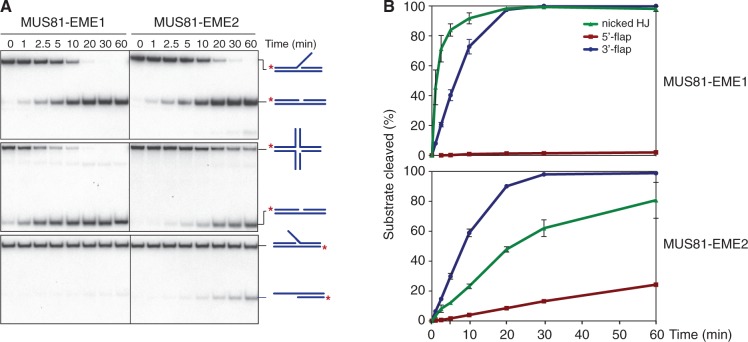
Cleavage of 3′-flap, 5′-flap and nicked HJs by MUS81-_SF_EME1 and MUS81-_SF_EME2. (**A**). The ^32^P-labelled DNA substrates (100 nM) were incubated with MUS81-EME1 (5 nM) or MUS81-EME2 (1 nM) and the products analysed by neutral PAGE. The 5′-^32^P-end labels are indicated with asterisks. (**B**). Product formation was quantified by phosphorimaging and expressed as a percentage of total radiolabelled DNA. The data presented are the mean of three independent experiments (±SEM).

**Figure 6.**
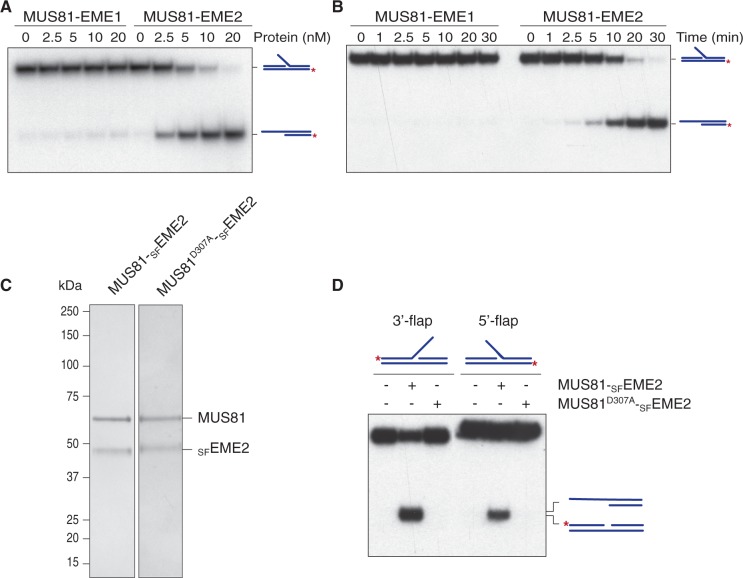
Cleavage of 5′-flap DNA by MUS81-EME2. (**A**). The ^32^P-labelled 5′-flap DNA (100 nM) was incubated with the indicated concentrations of MUS81-EME1 or MUS81-EME2 for 30 min at 37°C, and the products analysed by neutral PAGE and autoradiography. The 5′-^32^P-end labels are indicated with asterisks. (**B**) Time course using ^32^P-labelled 5′-flap DNA (100 nM) and 20 nM MUS81-EME1 or MUS81-EME2. (**C**). SDS–PAGE of purified MUS81-_SF_EME2 and catalytically inactive MUS81^D307A^-_SF_EME2, stained with InstantBlue. (**D**). The 5′-^32^P-end labelled 3′-flap and 5′-flap DNAs (100 nM) were incubated with purified MUS81-_SF_EME2A (2 nM) or catalytically inactive MUS81^D307A^-_SF_EME2A (2 nM) for 30 min at 37°C.

To determine whether the ability of MUS81-EME2 to cleave 5′-flaps might simply be a consequence of its higher specific activity than MUS81-EME1, we compared the actions of 5 nM MUS81-EME1 with 1 nM MUS81-EME2 (Figure 5A and B). We found that MUS81-EME2, but not the higher concentration of MUS81-EME1, retained the ability to cut the 5′-flap DNA. This distinction between MUS81-EME1 and MUS81-EME2 was even more apparent at high enzyme concentrations (Figure 6A and B). To confirm that the apparent 5′-flap endonuclease activity of MUS81-EME2 was intrinsic to the protein, catalytic-dead MUS81^D307A^-_SF_EME2 was purified using a similar scheme to that used for the wild-type protein (Figure 6C). Inactivation of MUS81 by site-directed alanine mutagenesis of the active site aspartate (D307A) has been reported previously (27). We observed that MUS81^D307A^-_SF_EME2 exhibited no endonuclease activity with either the 3′- or 5′-flap substrate (Figure 6D).

### Cleavage of 3′- and 5′-flap substrates by MUS81-EME2

**Figure 7.**
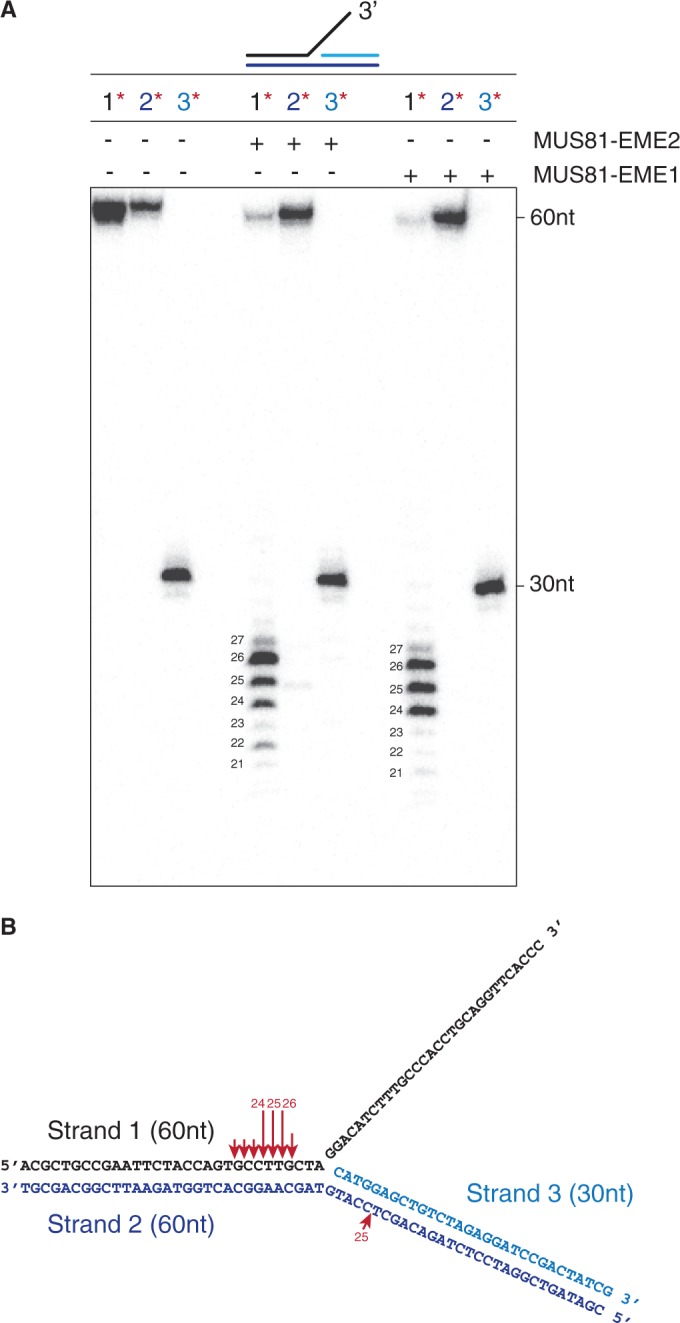
Cleavage of 3′-flap DNA by MUS81-_SF_EME1 and MUS81-_SF_EME2. (**A**). The 3′-flap DNA (100 nM), 5′-^32^P-labelled in strand 1, 2 or 3 as indicated (red asterisks), was incubated with purified MUS81-EME1 (5 nM) or MUS81-EME2 (2 nM) for 15 min at 37°C, and the reaction products were analysed by denaturing PAGE and autoradiography. The 60 and 30 nt indicate the uncut oligonucleotides. (**B**). Schematic representation of the 3′-flap substrate showing the sites of cleavage (red arrows) by MUS81-EME1 and MUS81-EME2A. Arrow size is proportional to the relative efficiency of cleavage.

Given that both MUS81-EME1 and MUS81-EME2 cut 3′-flaps efficiently, we next determined whether they exhibited the same pattern of incision. To do this, purified MUS81-EME1 and MUS81-EME2 were incubated with 3′-flap DNAs that were 5′-^32^P-end-labelled on strand 1, 2 or 3, and the reaction products were analysed by denaturing PAGE (Figure 7A). In this experiment, ^32^P-labelled marker oligonucleotides were used to determine the positions of the incisions (Figure 7, and data not shown). Both MUS81-EME1 and MUS81-EME2 cut the same region of DNA (5′-T^↓^G^↓^C^↓^C^↓^T^↓^T^↓^G^↓^C-3′), with the three major incisions occurring at the sequence 5′-C^↓^T^↓^T^↓^G-3′ (Figure 7B, red arrows). However, in contrast to MUS81-EME1, MUS81-EME2 also introduced a minor incision site into the strand opposite to that containing the flap, at the site 5′-CT^↓^CC-3′ (Figure 7A and B).

**Figure 8.**
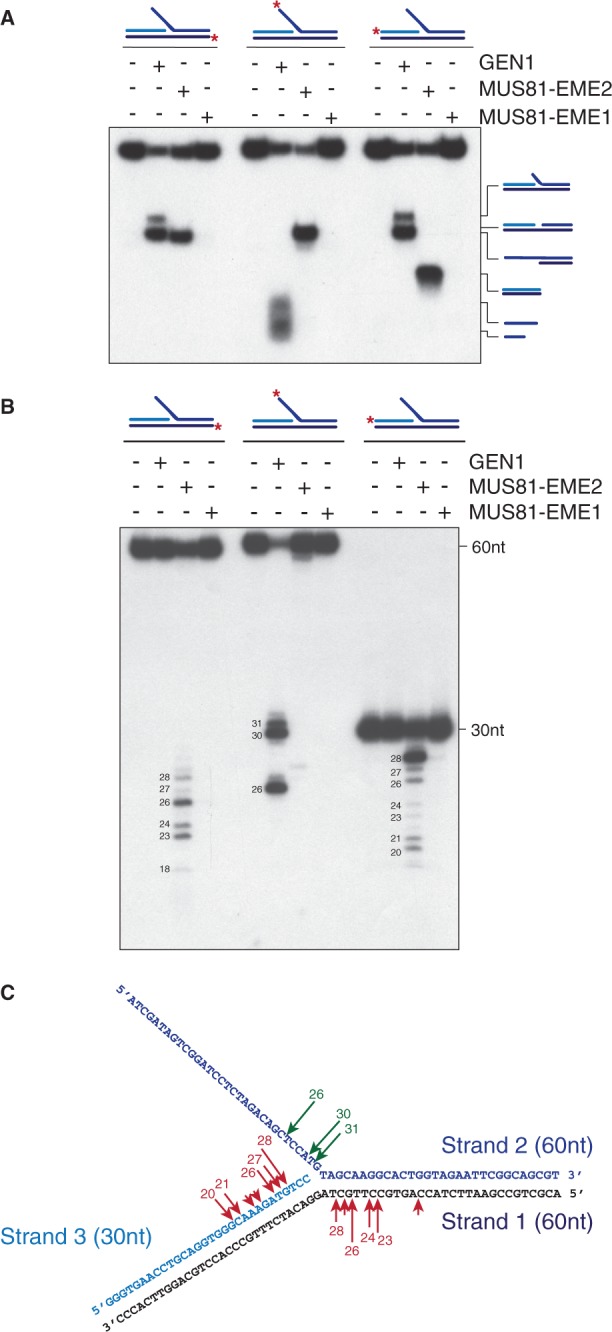
Cleavage of 5′-flap DNA by MUS81-_SF_EME2. The 5′-flap DNA (3 nM) was incubated with purified MUS81-EME1 (1 nM) or MUS81-EME2 (1 nM) for 30 min at 37°C. Purified GEN1^1–527^ (0.5 nM) was used as a positive control (10 min incubation at 37°C). Samples were divided in half and analysed by neutral (**A**) or denaturing PAGE (**B**). Red asterisks indicate ^32^P-labelled oligonucleotides. In (B), the sizes of intact and cleaved oligonucleotides are indicated. (**C**). Schematic representation of the cleavage sites introduced into the 5′-flap DNA by MUS81-EME2 (red arrows) or GEN1^1–527^ (green arrows). Arrow size is proportional to the relative efficiency of cleavage.

To gain further insights into the mechanism by which MUS81-EME2 cuts 5′-flaps, we analysed its activity with flap structures that were 5′-^32^P-end-labelled on strand 1, 2 or 3, and the DNA products were analysed by both neutral and denaturing PAGE (Figure 8A and B). As controls we used MUS81-EME1, which fails to cut this substrate, and a truncated version of the 5′-flap/HJ resolvase GEN1, GEN1^1^^−^^527^ (34,38). As expected, GEN1 removed the 5′-flap by cleaving strand 2 at one of three main sites: 5′-GC^↓^TCCA^↓^T^↓^GT-3′ (Figure 8B and C) (34). The pattern of cleavage produced by MUS81-EME2, however, was different from that of GEN1^1^^−^^527^. The junction was resolved by incisions that occurred in strand 1, such that a duplex arm (rather than the ssDNA flap) was removed. We also observed significant nicking in strand 3 (this may be due to ‘breathing’ or thermal denaturation of the resulting half-length gapped duplex). These results show that the apparent 5′-flap endonuclease activity of MUS81-EME2 relates to its ability to promote the removal of a duplex arm, close to the flap, rather than the single-stranded 5′-flap itself.

### Resolution of recombination intermediates by MUS81-EME2

**Figure 9.**
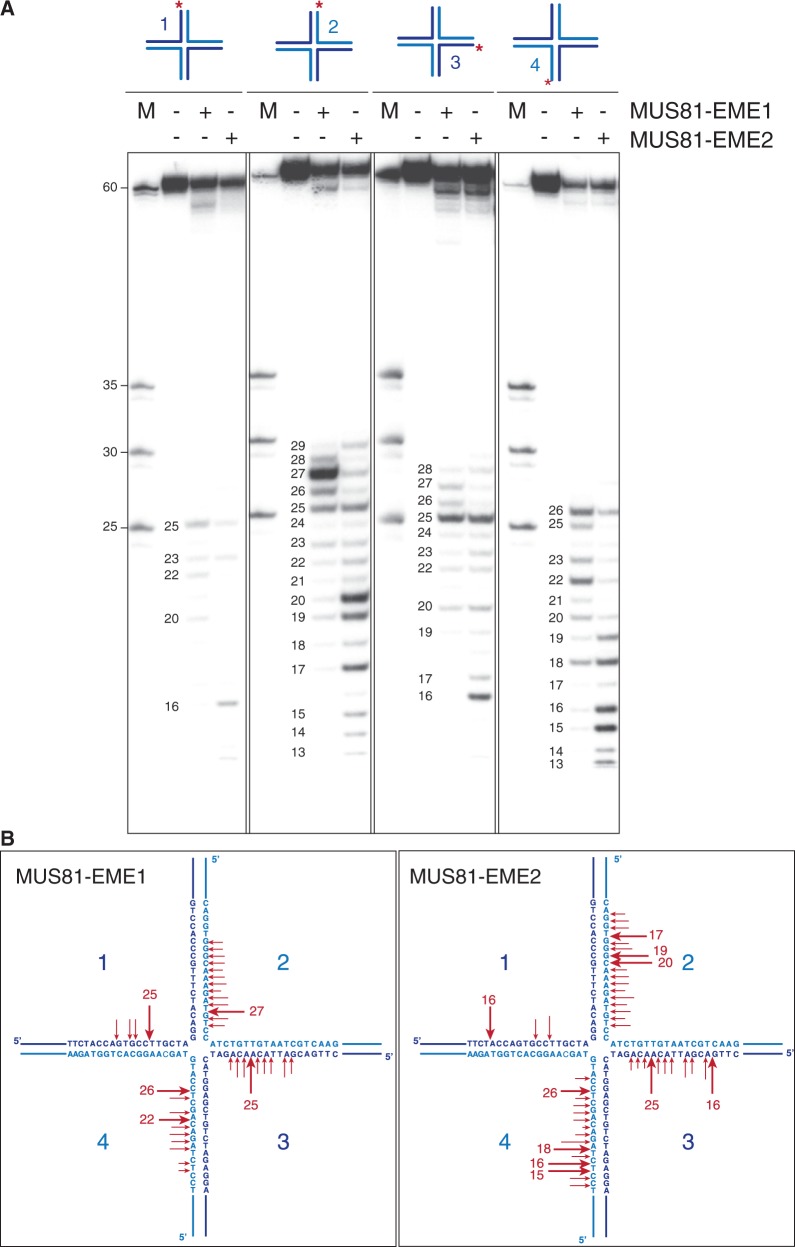
Holliday junction cleavage by MUS81-EME2. (**A**) An immobile HJ (∼3 nM), 5′-^32^P-end-labelled on strands 1, 2, 3 or 4 (as indicated by red asterisks), was incubated with purified MUS81-_SF_EME1 (5 nM) or MUS81-_SF_EME2 (1 nM) for 30 min at 37°C. Reaction products were analysed by denaturing PAGE. The sizes of the cleavage fragments are indicated. M = 5′-^32^P-end-labelled size markers. (**B**). Schematic representation of the HJ showing sites of incision (red arrows) by either MUS81-EME1 or MUS81-EME2. Arrow size is proportional to the relative efficiency of the cleavage.

Next, we analysed the actions of MUS81-EME2 on HJs and D-loops, two forms of recombination intermediates. Earlier we showed that MUS81-EME2 cleaved HJs more efficiently than an equivalent concentration of MUS81-EME1 (Figure 4B), although this is not a preferred substrate for either nuclease. To determine the sites of weak cleavage, MUS81-EME1 and MUS81-EME2 were incubated with immobile HJs that were 5′-^32 ^P-end-labelled on strand 1, 2, 3 or 4, and the products were analysed by denaturing PAGE (Figure 9). We found that both MUS81 complexes introduced multiple incisions in all four strands of the HJ (Figure 9B). In contrast to a canonical HJ resolvase such as GEN1, the incisions occurred in an asymmetric manner. With this particular immobile HJ, cleavage occurred preferentially in strands 2 and 4, most likely due to the conformation of the junction, and the nicks introduced by MUS81-EME1 tended to be closer to the branch point compared with those made by MUS81-EME2.

**Figure 10.**
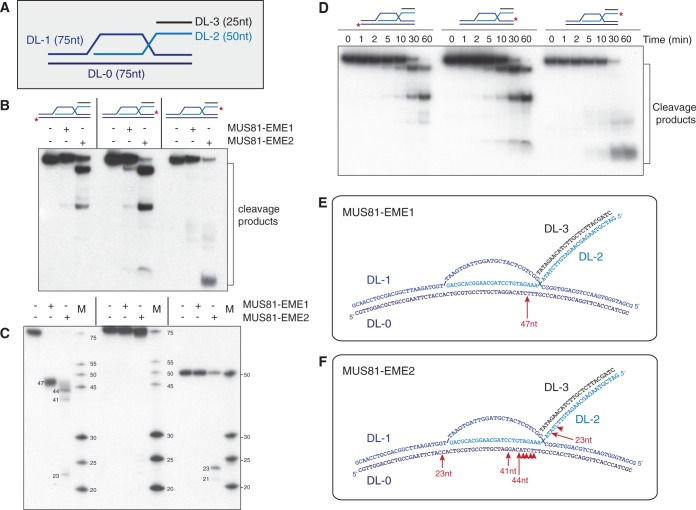
Cleavage of D-loop structures by MUS81-_SF_EME1 and MUS81-_SF_EME2. (**A**) Schematic representation of the D-loop structure, indicating the strands and oligo lengths. (**B** and **C**). D-loop DNA (100 nM), 5′-^32^P-end-labelled on strands DL-0, DL-1 or DL-2 (as indicated by red asterisks), was incubated with purified MUS81-EME1 (5 nM) or MUS81-EME2A (5 nM) for 30 min at 37°C. Samples were divided in half and analysed by neutral (B) or denaturing PAGE (C). The sizes of intact and cleaved oligonucleotides are indicated. M indicates 5′-^32^P-end-labelled size marker oligonucleotides. (**D**). Time course of the cleavage of D-loop DNA (100 nM), 5′-^32^P-end-labelled on strands DL-0, DL-1 or DL-2 (as indicated by red asterisks), by MUS81-_SF_EME2 (5 nM). Incubation was at 37°C for the indicated times, and products were analysed by neutral PAGE and autoradiography. (**E** and **F**). Schematic representation of the D-loop showing sites of cleavage by MUS81-EME1 or MUS81-EME2, respectively. Arrow size is proportional to the relative efficiency of cleavage.

Finally, we compared the actions of MUS81-EME1 and MUS81-EME2 on D-loop structures. To do this, equal amounts of the purified proteins were incubated with D-loop structures that were 5′-^32^P-end-labelled on strands DL-0, DL-1 or DL-2 (Figure 10A). We observed that MUS81-EME2 was ∼10-fold more efficient in promoting D-loop cleavage than MUS81-EME1, as analysed by neutral PAGE (Figure 10B). The cleavage sites were mapped by denaturing PAGE, revealing that MUS81-EME1 performed only a single incision and this occurred on the strand complementary to the invading single strand, four nucleotides away from the junction point (Figure 10C and E). In contrast, MUS81-EME2 introduced multiple cuts around the junction point and additionally introduced a single nick at the other side of the D-loop at a position complementary to the 3′-invading end (Figure 10C and F). These products arose simultaneously, as indicated in the time course experiment (Figure 10D), showing that MUS81-EME2 processes D-loop structures using a one-step reaction. Thus, in contrast to MUS81-EME1, MUS81-EME2 disengages the D-loop structure by cleaving the 3′-invading strand.

## DISCUSSION

In this study, we report the identification of two isoforms of human EME2, referred to as EME2 and EME2B. Both isoforms align mainly with the C-terminal region of EME1 and interact with endogenous MUS81. Like MUS81-EME1, MUS81-EME2 and MUS81-EME2B have a single subunit (MUS81) with an active ERCC4 nuclease domain because, as already reported for EME1, the ERKXXXD catalytic motif has evolutionarily diverged in both EME2 and EME2B (26). As we found little evidence for the expression of EME2B in human cells, we compared the endonucleolytic activities of purified MUS81-_SF_EME1 and MUS81-_SF_EME2 on a set of synthetic model DNA structures. We found that the interaction between MUS81 and EME2 results in the formation of a novel 3′-flap endonuclease, which differs from MUS81-EME1 in both efficiency and mechanism of cleavage with a variety of model DNA substrates. Specifically, we found that MUS81-EME2 was ∼5-fold more active than MUS81-EME1 on all DNA substrates analysed and, in contrast to MUS81-EME1, it was able to process a 5′-flap structure by cleaving the DNA strand complementary to that containing the flap. As such, it removes a duplex DNA arm, in a reaction not seen with MUS81-EME1.

The mechanisms by which MUS81-EME1 and MUS81-EME2 cleaved a 3′-flap were similar: both complexes cut within the duplex DNA region 3–7 nt on the 5′-side of the branch point. This cleavage pattern is consistent with that observed with purified *S. cerevisiae* Mus81-Mms4, which binds to the 5′-end located downstream of the flap and cleaves the duplex DNA 3–7 nt upstream of the branch point (24). Hence, it is likely that the cleavage mechanism of 3′-flap structures is an evolutionarily conserved feature of the MUS81 endonuclease, which does not depend on the identity of the catalytically inactive subunit. Nevertheless, we observed that, unlike MUS81-EME1, MUS81-EME2 was able to process the 3′-flap in a two-step reaction: in the first step, which is comparable with that mediated by MUS81-EME1, MUS81-EME2 removes the flap, generating a nicked duplex molecule; in the second step, it introduces a minor cleavage opposite the nick, thus generating smaller duplex DNA products. MUS81-EME2 uses the same mechanism to process replication forks, but the biological relevance of the second reaction step is presently unknown.

MUS81-EME1 and MUS81-EME2 differ in the cleavage mechanism of recombination intermediate structures such as D-loops and HJs. D-loops are intermediate structures of DSB repair, and related structures (T-loops) are found at telomeres when 3′-G-rich single-stranded overhangs invade duplex telomeric DNA (39,40). When D-loops were exposed to the activities of MUS81-EME1 and MUS81-EME2, we observed that MUS81-EME2 was able to cleave the invading strand 2 and 4 nt on the 5′-side of the invasion point, thereby disengaging the D-loop. Such reactions were not observed with MUS81-EME1. Additionally, when we compared their ability to cleave intact HJs, we found that the major cuts inserted by MUS81-EME1 were closer to the branch point than those introduced by MUS81-EME2.

Taken together, these results indicate that MUS81-EME1 and MUS81-EME2 are 3′-flap endonucleases that differ in cleavage efficiency and substrate preference. We found that MUS81-EME2 was more active than MUS81-EME1, and it preferentially cleaved 3′-flaps and RFs, whereas the nicked HJ was the preferred substrate for MUS81-EME1. Such differences in substrate specificity might reflect rather different functional roles for MUS81-EME1 and MUS81-EME2 *in vivo*.

EME1 is the regulatory subunit of MUS81-EME1. Its phosphorylation by CDK, and to a lesser extent by PLK1, at G2/M contributes to the formation of the SLX-MUS complex, which promotes HJ resolution and is necessary for proper chromosome segregation (9). The precise biological role of MUS81-EME2 in human cells is presently unknown, although its relocalization within the nucleus in response to DNA damage is suggestive of a role in DNA repair. One intriguing possibility is that EME2 contributes to the regulation of the MUS81-EME2 endonuclease. In this way, MUS81-EME2 activity might be modulated in a way that is distinct from that of MUS81-EME1, and thus the two nucleases may be specifically targeted to distinct DNA substrates at different stages of the cell cycle. Future studies will focus on determining the biological roles of MUS81-EME2.

## FUNDING

Cancer Research UK, the European Research Council, the Louis-Jeantet Foundation, the Swiss Bridge Foundation and the Breast Cancer Campaign. Funding for open access charge: European Research Council.

*Conflict of interest statement*. None declared.
